# NS2 is dispensable for efficient assembly of hepatitis C virus-like particles in a bipartite *trans*-encapsidation system

**DOI:** 10.1099/vir.0.068932-0

**Published:** 2014-11

**Authors:** Matthew J. Bentham, Najat Marraiki, Christopher J. McCormick, David J. Rowlands, Stephen Griffin

**Affiliations:** 1 Leeds Institute of Cancer & Pathology (LICAP), and Leeds CRUK Clinical Centre, Faculty of Medicine and Health, St James’s University Hospital, University of Leeds, Beckett St., Leeds, West Yorkshire LS9 7TF, UK; 2 School of Molecular & Cellular Biology and Astbury Centre for Structural Molecular Biology, Faculty of Biological Sciences, University of Leeds, Mount Preston Street, Leeds, West Yorkshire LS2 9JT, UK; 3 Faculty of Medicine, University of Southampton, Southampton General Hospital, Tremona Road, Southampton SO16 6YD, UK

## Abstract

Infectious hepatitis C virus (HCV) particle production in the genotype 2a JFH-1-based cell culture system involves non-structural proteins in addition to canonical virion components. NS2 has been proposed to act as a protein adaptor, co-ordinating the early stages of virion assembly. However, other studies have identified late-acting roles for this protein, making its precise involvement in infectious particle production unclear. Using a robust, bipartite *trans*-encapsidation system based upon baculovirus expression of HCV structural proteins, we have generated HCV-like particles (HCV-LP) in the absence of NS2 with overt similarity to wild-type virions. HCV-LP could transduce naive cells with *trans*-encapsidated subgenomic replicon RNAs and shared similar biochemical and biophysical properties with JFH-1 HCV. Both genotype 1b and JFH-1 intracellular HCV-LP were produced in the absence of NS2, whereas restoring NS2 to the JFH-1 system dramatically enhanced secreted infectivity, consistent with a late-acting role. Our system recapitulated authentic HCV particle assembly via *trans*-complementation of bicistronic, NS2-deleted, chimeric HCV, which is otherwise deficient in particle production. This closely resembled replicon-mediated NS2 *trans*-complementation, confirming that baculovirus expression of HCV proteins did not unduly affect particle production. Furthermore, this suggests that separation of structural protein expression from replicating HCV RNAs that are destined to be packaged alleviates an early stage requirement for NS2 during particle formation. This highlights our current lack of understanding of how NS2 mediates assembly, yet comparison of full-length and bipartite systems may provide further insight into this process.

## Introduction

Hepatitis C virus (HCV) represents a global health challenge with a prevalence of over 3 % worldwide. Chronic infection leads to severe liver disease in many cases, including cirrhosis and hepatocellular carcinoma. Despite the development of an HCV infectious culture system based on the genotype 2a JFH-1 isolate (Kato *et al.*, 2003; Wakita *et al.*, 2005), the way in which infectious particles are both assembled and secreted from infected cells remains poorly characterized.

HCV is an enveloped virus with a 9.6 kb positive sense ssRNA genome belonging to the *Flaviviridae*. An internal ribosome entry site (IRES) present within the 5′ UTR drives translation of a ~3000 aa polyprotein that is cleaved by both host and viral proteases yielding 10 mature products. The core (C) and envelope proteins (E1 and E2) along with the RNA genome are thought to be the major virion components, whilst the non-structural (NS) proteins 3, 4A, 4B, 5A and 5B are both necessary and sufficient to form the viral RNA replication complex. Two proteins, p7 and NS2, do not easily fall within either category.

It is increasingly clear that HCV non-structural proteins co-operate with the canonical virion components during infectious particle production. NS2 has been proposed to act both at early and late times during this process. The early role for NS2 is thought to involve its ability to act as a protein adaptor (Jirasko *et al.*, 2008, 2010; Ma *et al.*, 2011; Stapleford & Lindenbach, 2011), meditating interactions between both structural (e.g. E2) and other non-structural proteins (e.g. NS3, NS5A). This function is, in turn, dependent on a genotype-specific interaction with p7, which targets NS2 to punctae within infected cells (Jirasko *et al.*, 2010; Popescu *et al.*, 2011; Tedbury *et al.*, 2011), and the two proteins together also modulate core protein localization (Boson *et al.*, 2011). Co-localization analyses show that NS2 punctae contain other HCV non-structural proteins such as NS5A, which, complemented by interaction data, have led to the proposal that they represent sites of virion assembly. Accordingly, point mutations that disrupt these interactions also prevent punctae formation and abrogate intracellular infectivity, although whether non-infectious particles are formed within cells is not known.

Evidence for a late-acting role for NS2 includes detection of non-infectious core-containing intracellular particles in cells harbouring chimeric H77/JFH-1 HCV with a deleterious NS2 S168A mutation (Yi *et al.*, 2009). Restoration of infectivity in this context was achieved by NS2 expression *in trans*, and was also genotype dependent. More recently, studies of the NS2 N-terminal region identified a subset of point mutants with particle production defects specific to virion egress rather than assembly; accumulation of intracellular infectivity was unaffected (de la Fuente *et al.*, 2013). Thus, NS2 appears to play a complex role during virion morphogenesis, potentially involving more than one essential function.

It is well established that the canonical HCV structural proteins (core, E1, E2), expressed in multiple contexts, can form HCV-like particles (HCV-LP) that physically resemble wild-type HCV (wtHCV) (Baumert *et al.*, 1998; Kunkel *et al.*, 2001; Owsianka *et al.*, 2001; Saunier *et al.*, 2003; Triyatni *et al.*, 2002; Wellnitz *et al.*, 2002; Xiang *et al.*, 2002). Thus, *de novo* capsid formation and envelopment are not dependent on NS2 in these systems. However, further studies report a dependence on NS2 for the formation of secreted HCV-LP capable of delivering packaged replicon RNA to naive cells: so-called pseudo-infection (Adair *et al.*, 2009; Ishii *et al.*, 2008; Steinmann *et al.*, 2008). This leaves the intriguing possibility that, in the absence of NS2, the intracellular compartment harbours HCV-LP produced by *trans*-encapsidation that are capable of pseudo-infection.

Here, we show that functional intracellular HCV-LP capable of pseudo-infection are generated by a bipartite *trans*-encapsidation system lacking NS2. HCV-LP resembling particles produced by full-length HCV clones were formed by both genotype 1b and a more vigorous genotype 2a JFH-1 system. Reintroduction of NS2 into the JFH-1 context led to a significant increase in secreted infectivity, consistent with the restoration of a late-acting function in this *trans*-encapsidation setting.

## Results

### NS2 is dispensable for intracellular HCV-LP formation by genotype 1b HCV

**Fig. 1.**
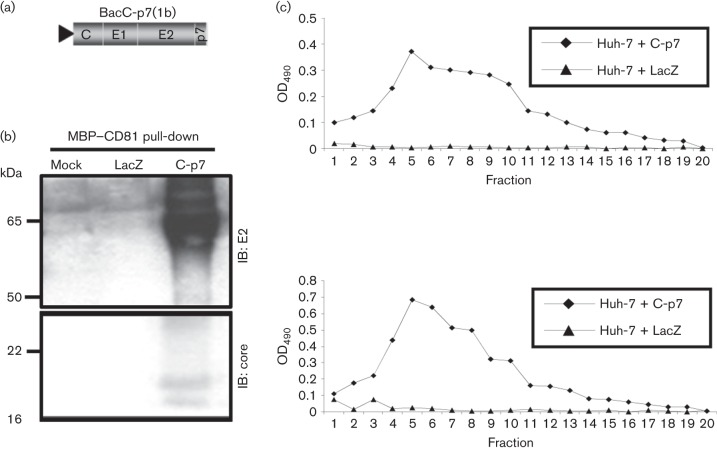
Formation of intracellular particles in GT1b system lacking NS2. (a) Diagram of genotype 1b C-p7 baculovirus construct. (b) MBP–CD81 pull-down from detergent-free lysate derived from transduced Huh7 cells as indicated. MBP–CD81 was incubated with lysates and the complexes were pulled down on amylose beads. Bound material was assayed for the presence of E2 and core by immunoblot (IB). (c) Top graph, indirect ELISA for MBP–CD81 performed on fractionated gradients of detergent-free BacC-p7/Huh7 lysates. BacC-p7/Huh7 lysates were partitioned on sucrose gradients, which were subsequently fractionated and 100 *μ*l of each fraction was bound to one well of an ELISA plate. Post-washing, each was incubated with MBP–CD81, which was subsequently detected with rabbit anti-MBP, followed by goat anti-rabbit-HRP. Bottom graph, MBP–CD81 was bound to the ELISA plate, which was subsequently incubated with 100 *μ*l of each fraction. Detection was carried out using mouse anti-E2 (AP33) followed by anti-mouse-HRP. ⧫, Huh7 + Cp7; ▴, Huh7 + LacZ.

We first examined whether ‘empty’ HCV-LP, reminiscent of those produced in Sf9 cell studies (Baumert *et al.*, 1998), could be generated by transduction of Huh7 cells with a baculovirus encoding a genotype 1b C-E1-E2-p7 expression cassette [BacC-p7(1b)] (Fig. 1a). To demonstrate biochemically the presence of enveloped particles, detergent-free lysates prepared from transduced Huh7 cells were incubated with recombinant CD81 large extracellular loop (LEL) fused to maltose binding protein (MBP–CD81) in order to precipitate HCV-LP via the E2/CD81-receptor/ligand interaction (Chan-Fook *et al.*, 2000). Western blotting of bound fractions demonstrated the presence of both E2 and core in lysates transduced by BacC-p7(1b), but not a Bac-LacZ control (Fig. 1b). HCV proteins were not detectable in supernatants (data not shown). Next, lysates were partially purified through a sucrose cushion and then separated by sucrose gradient ultracentrifugation. MBP–CD81 was used either as a probe for E2-containing fractions bound to an ELISA plate (Fig. 1c, top graph), or to coat plates and serve as an immobilized trap for E2 within gradient fractions (Fig. 1c, bottom graph). In each case, a broad peak of reactivity absent from controls was detected, consistent with particulate material partitioning within the density gradients.

**Fig. 2.**
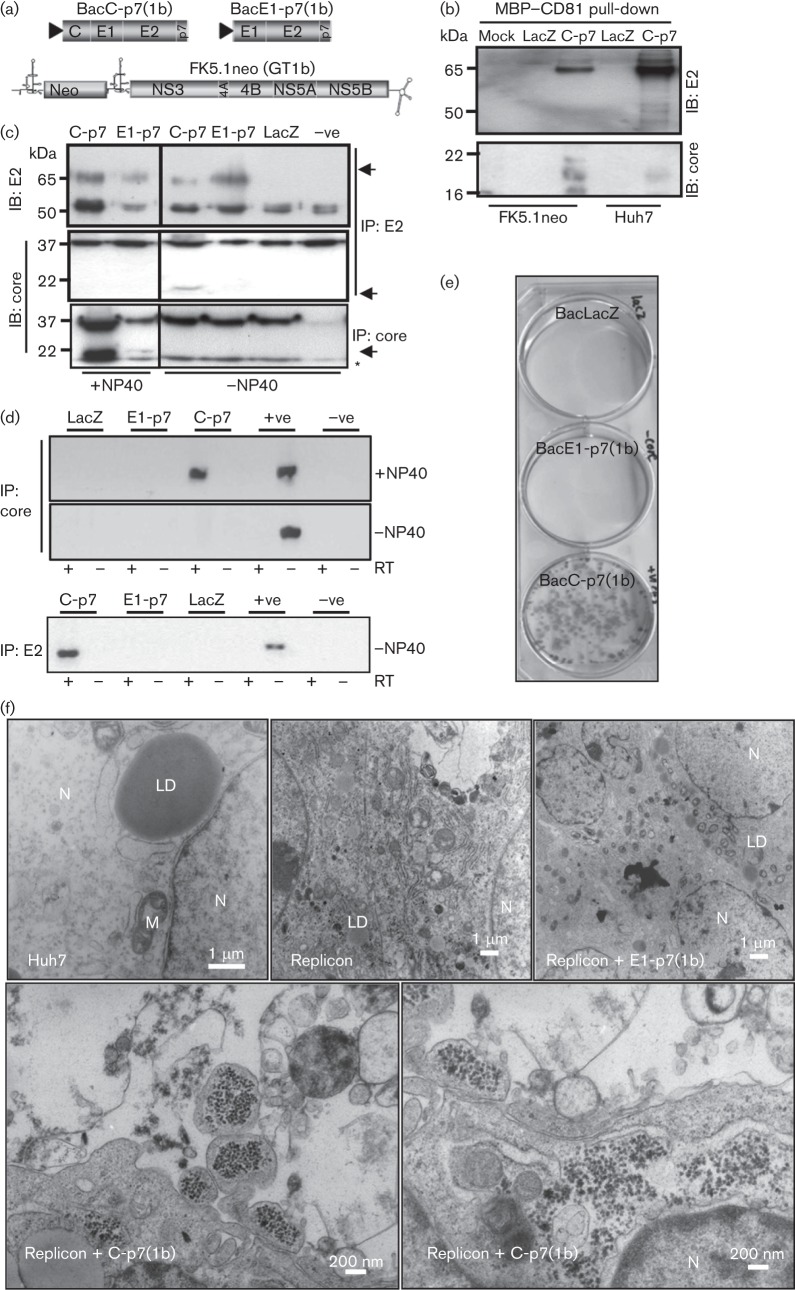
GT1b particles form in the absence of NS2, package replicon RNA and pseudo-infect naive Huh7 cells. (a) Diagram of genotype-1b-expressing baculoviruses and the FK5.1neo replicon. (b) MBP–CD81 pull-down from FK5.1 replicon-containing cells transduced with baculoviruses as indicated. Pull-downs were blotted for either E2 or core. (c) Immunoprecipitation of HCV material from FK5.1neo cell lysates, transduced with baculoviruses as indicated, in the presence/absence of 0.5 % NP-40 detergent. IP, immunoprecipitation; IB, immunoblot; asterisk denotes non-specific band detected by anti-GT1b core antibody; arrows highlight band of interest for each immunoblot. (d) HCV-specific reverse transcriptase (RT)-PCR on material immunoprecipitated as described for (c). RT, reverse transcriptase; +ve, *in vitr*o transcribed replicon RNA positive control; −ve, water negative control. (e) Huh7 colonies selected with G418 (1 mg ml^−1^) following pseudo-infection with replicon cell lysates post-transduction with baculoviruses as indicated. (f) Thin section transmission electron microscopy of Huh7 or FK5.1neo cells transduced with baculoviruses as indicated. N, nucleus; M, mitochondrion; LD, lipid droplet; bars as indicated (calculated).

Next, we investigated whether intracellular HCV-LP could encapsidate replicon RNA and mediate pseudo-infection using a bipartite *trans*-encapsidation system employing baculovirus transduction [BacC-p7(1b)] of Huh7 cells carrying the culture-adapted FK5.1neo replicon (Krieger *et al.*, 2001) encoding NS3-5B (Fig. 2a). MBP–CD81 pull-downs from detergent-free lysates of BacC-p7(1b)-transduced Huh7 or Huh7 FK5.1neo cells demonstrated the presence of correctly folded E2 in close association with core protein (Fig. 2b), suggestive of particle formation.

The composition and structure of potential HCV-LP was next investigated by immunoprecipitation. Consistent with the presence of intact enveloped virions, detergent disrupted the ability of anti-E2 antibodies to precipitate either core protein (Fig. 2c) or replicon RNA (Fig. 2d). Similarly, anti-E2 antibodies did not precipitate replicon RNA from cells transduced with a baculovirus lacking the core protein, BacE1-p7(1b), excluding the possibility of exosomal transfer of replicon RNAs (Fig. 2c). In addition, replicon RNA was only precipitated by anti-core antibodies in the presence of detergent, excluding non-enveloped capsids from mediating RNA transfer (Fig. 2d).

HCV-LP pseudo-infection was then assessed by G418-resistant colony formation in naive Huh7 cells transduced with either secreted or intracellular HCV-LP preparations. Each experiment was conducted upon the same batch of the replicon-bearing stable cell line, ensuring equivalent RNA copies per cell between transductions. Whilst pseudo-infectivity was absent from the supernatants of BacC-p7(1b)-transduced Huh7 FK5.1neo cells (data not shown), intracellular infectivity released by detergent-free lysis was readily detectable by colony formation assay (Fig. 2e). This was not the case for control baculoviruses BacLacZ or BacE1-p7(1b). Furthermore, selected individual colonies expanded under selection were shown to maintain replicon RNA by Northern blot (data not shown).

The presence of intracellular HCV-LP within producer cells was examined by thin section transmission electron microscopy (TEM) (Fig. 2f). Membrane rearrangements were evident within replicon cells, including those transduced with BacE1-p7(1b), yet particles were only evident within cells both harbouring replicon RNA and transduced by BacC-p7(1b). Interestingly, these particles were concentrated within distended intracellular vesicles, consistent with a potential block to secretion. On the basis of evidence from immunoprecipitation experiments, we attest that electron-dense entities are likely to represent HCV-LP, assembled in the absence of NS2.

**Fig. 3.**
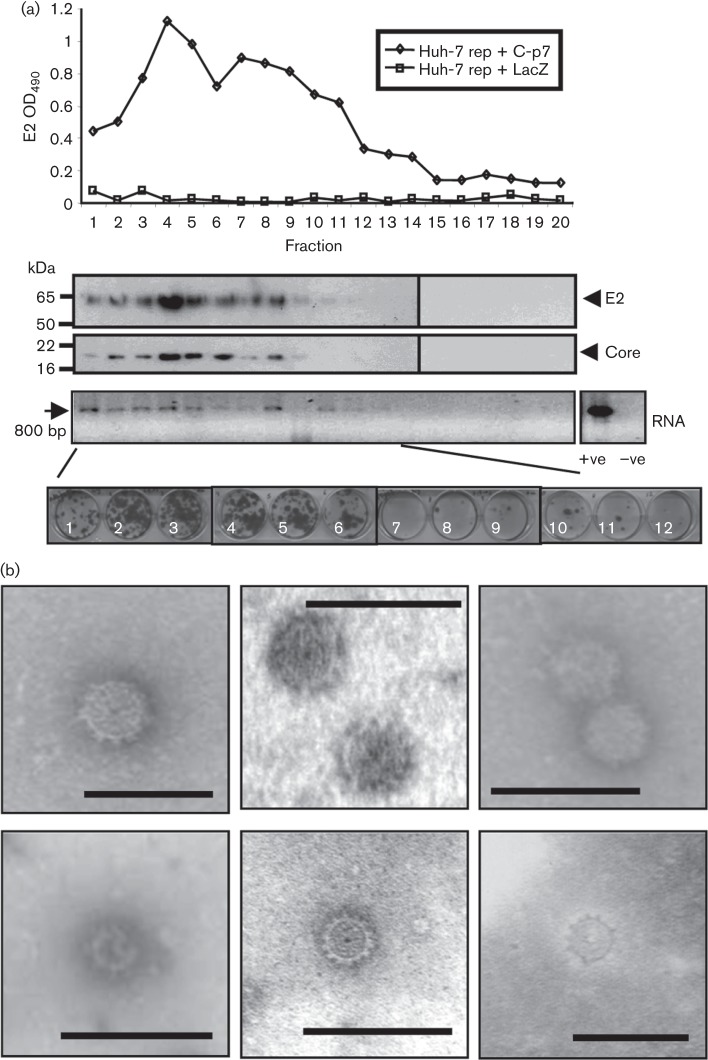
Direct analysis of intracellular HCV-LP on sucrose density gradients. (a) Anti-E2 ELISA using ALP98 mouse monoclonal antibody conducted on fractions from a 10–60 % (w/v) sucrose density gradient from C-p7-transduced FK5.1neo cell lysates. Western blots for HCV proteins and RT-PCR for HCV RNA are shown underneath for each fraction, along with colony formation assays for the top 12 fractions. (b) Negative-stain TEM of peak gradient fraction (4) revealing HCV-LP. Bars, 100 nm.

Intracellular RNA-containing HCV-LP were next analysed by sucrose density gradient ultracentrifugation (Fig. 3a). HCV E2 ELISA indicated a broad peak of particulate material towards the top of the gradient, which we showed to contain E2 and core (by Western blot), replicon RNA [by reverse transcriptase (RT)-PCR], and pseudo-infectivity (colony formation). Furthermore, TEM analysis of peak gradient fractions revealed the presence of enveloped particles with diameters of around 50 nm (Fig. 3b). Some HCV-LP contained a clearly identifiable core as well as prominent envelope spike proteins. Equivalent particles were not observed in control gradients from LacZ-transduced cells, or within non-peak fractions from the BacC-p7(1b)-transduced cells (data not shown). Thus, pseudo-infectious genotype 1b HCV-LP assemble within cells in the absence of NS2 but are not secreted, and are reminiscent of particles produced in analogous systems as well as authentic wtHCV virions.

### JFH-1 HCV-LP are assembled, but not efficiently secreted in the absence of NS2

**Fig. 4.**
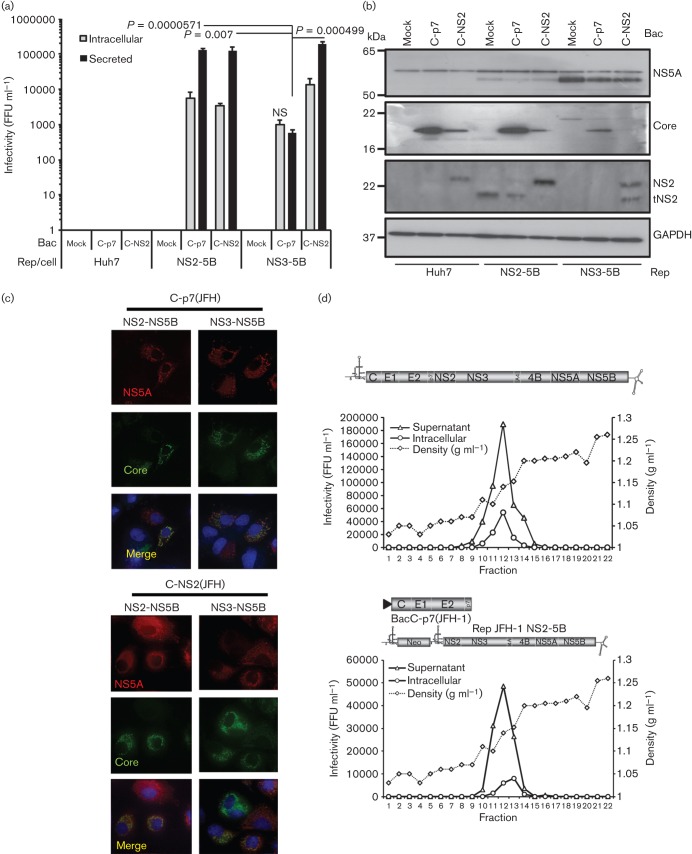
Infectivity and analysis of JFH-1 HCV-LP produced in the presence/absence of NS2. (a) Top, infectivity (focus forming units ml^−1^, FFU ml^−1^) of Huh7 lysates and supernatants from Huh7 cells electroporated with replicon RNA (Rep) and transduced with baculoviruses (Bac) as indicated. Results are the mean of at least three separate experiments with three wells in each. *P* values were generated using Student’s *t*-test. Bottom, expression of replicon (NS5A)- and baculovirus (core)-derived proteins along with GAPDH loading control. (b) Immunoblot showing expression of NS2 in relation to core and NS5A in transduced, replicon-expressing Huh7 cells. (c) Indirect immunofluorescence of replicon (NS5A)- and baculovirus (core)-derived proteins. NS5A is shown in the red channel (594 nm), core in green (488 nm) and nuclei counter-stained using Hoechst. (d) Infectivity profile of intracellular and secreted particles following isopycnic density gradient ultracentrifugation in a 10–40 % iodixinol/PBS gradient. Samples were processed at 72 h post electroporation. Top, JFH-1; bottom, HCV-LP derived using baculovirus BacC-p7(JFH-1)-transduced Huh7 cells electroporated with JFH-1 NS2-5B replicon RNA.

Given our observation that intracellular genotype 1b HCV-LP could form in the absence of NS2, we examined whether this was also the case in an analogous JFH-1 bipartite system, assessing HCV-LP pseudo-infectivity by focus-forming assay. Consistent with genotype 1b experiments, transduction of Huh7 cells transiently electroporated with the JFH-1 NS3-5B replicon with BacC-p7(2a) or BacC-NS2(2a) revealed no statistically significant differences (*P* = 0.082554) in pseudo-infectivity within cell lysates lacking NS2, compared with those where NS2 was present (Fig. 4a). Pseudo-infectivity did not directly correlate with levels of baculovirus-expressed protein; BacC-NS2(2a) showed markedly reduced expression levels compared with BacC-p7(2a) as noted previously (Adair *et al.*, 2009; Bentham *et al.*, 2013) (Fig. 4b). Low levels of secreted pseudo-infectivity were present for transductions lacking NS2, yet inclusion of NS2 had a profound effect on pseudo-infectivity within the secreted compartment, with highly significant (>2 log_10_, *P*<0.0005) increases in titre (Fig. 4a). Interestingly, levels of pseudo-infectivity appeared to be independent of whether NS2 was present upon the replicon or the baculovirus, despite low expression levels of BacC-NS2(2a). Furthermore, Western blot analysis of NS2 revealed a high propensity for NS2 to exist in its truncated (t) form where p7 was absent (Fig. 4b). This may relate to a requirement for p7 to stabilize NS2 resulting from their *trans*-interaction. Indirect immunofluorescence for core and NS5A confirmed the presence of cells co-expressing both replicon and baculovirus proteins in each set of conditions (Fig. 4c).

### JFH-1 HCV-LP resemble infectious JFH-1 wtHCV

To show that HCV-LP resembled infectious JFH-1 particles, we partially purified either clarified supernatants or detergent-free cell lysates through sucrose cushions prior to comparing buoyant density/infectivity profiles following centrifugation to equilibrium through isopycnic iodixinol density gradients (Fig. 4d). HCV-LP produced by a combination of BacC-p7(2a) and the NS2-NS5B replicon displayed a near-identical profile to that of JFH-1, with peak infectivity ranging from ~1.1 to 1.15 g ml^−1^. As for JFH-1, the majority of HCV-LP infectivity was present within the secreted compartment.

**Fig. 5.**
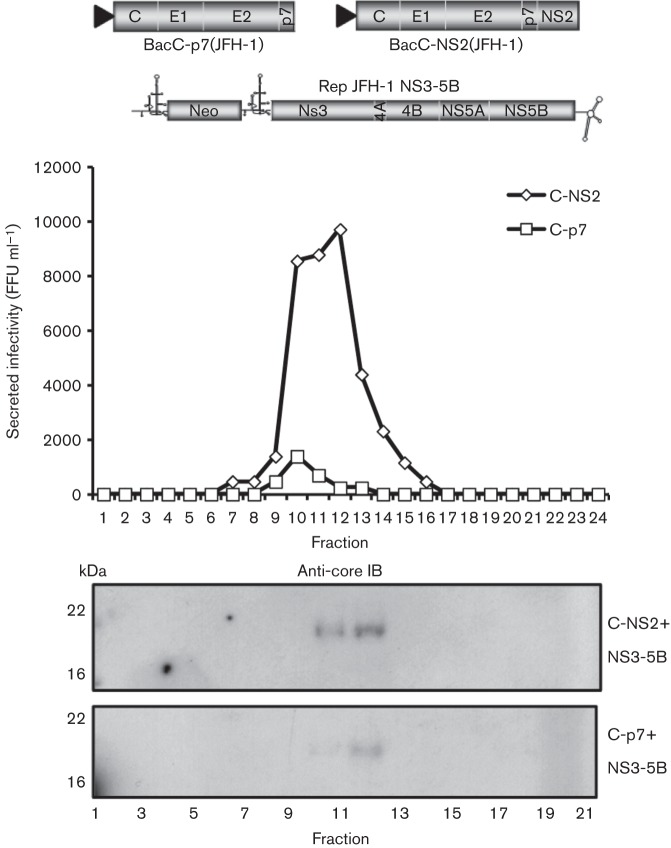
Characterization of secreted HCV-LP produced in the presence/absence of NS2. Top, infectivity profile of secreted HCV-LP generated in the absence/presence of NS2 (see diagram) following isopycnic density gradient ultracentrifugation in a 10–40 % iodixinol/PBS gradient. Bottom, core immunoblot of methanol-precipitated gradient fractions.

Consistent with bulk infectivity measurements (Fig. 4a), levels of secreted infectious HCV-LP produced in the absence of NS2 were much lower than those formed in its presence (Fig. 5, top panel). Nevertheless, core protein was detectable in the peak fractions from gradients of particles formed in the absence of NS2 (Fig. 5, lower panel). However, the reduction in protein levels was not proportionate to the loss of infectivity, suggesting that the specific infectivity of secreted HCV-LP may have been affected. It is also not possible to rule out that HCV-LP present within the secreted compartment result from cell lysis liberating particles that might not otherwise be efficiently secreted.

### Characterization of intracellular JFH-1 HCV-LP formed in the absence of NS2

**Fig. 6.**
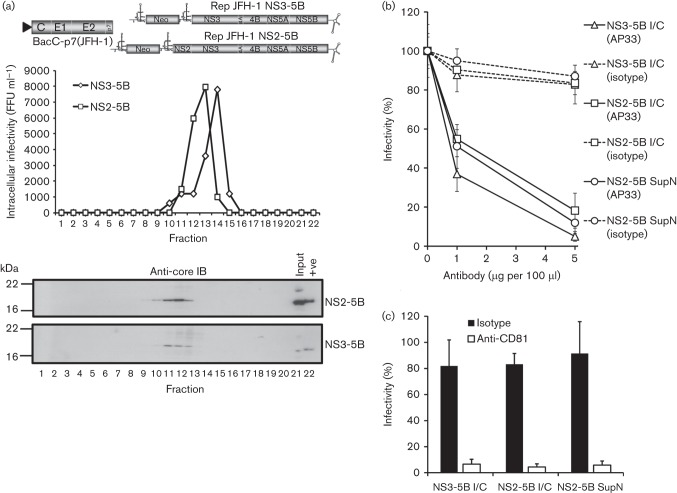
Characterization of HCV-LP produced in the presence/absence of NS2. (a) Comparison of intracellular HCV-LP infectivity profiles produced in the presence/absence of NS2 (see diagram) following isopycnic density gradient ultracentrifugation in a 10–40 % iodixinol/PBS gradient. Core immunoblots of methanol-precipitated gradient fractions are shown below. (b) Titration of HCV-LP infectivity using AP33-neutralizing monoclonal anti-E2 antibody. Huh7 cells plated into wells of a 96-well culture dish were infected with serially diluted HCV-LP supernatant (100 *μ*l) in the presence of increasing antibody concentration (AP33 or isotype control), as indicated. Antibody was pre-mixed with supernatants for 30 min prior to infection. (c) As for (b), but in this case cells were pre-incubated with anti-CD81 antibody (10 *μ*g ml^−1^) or isotype control. I/C, intracellular; SupN, supernatant.

We next compared intracellular JFH-1 HCV-LP produced in the presence/absence of NS2. Detergent-free lysates of transfected/transduced Huh7 cells were purified through a sucrose cushion, and then subjected to iodixinol gradient analysis as above. A peak of infectivity was present near the centre of the gradient (Fig. 6a, top panel), although particles formed in the absence of NS2 displayed a slightly higher peak buoyant density (~1.2 compared with 1.15 g ml^−1^). Similarly, peak fractions contained core protein in relative proportion to respective loading controls, indicating that organization of core into capsid structures was not adversely affected by the lack of NS2.

Critically, HCV-LP produced in the absence of NS2 were equally sensitive to antibody-mediated blockade of virus entry compared with those generated when the protein was present. Both titration of anti-E2 (AP33, Fig. 6b) and blockade of cellular CD81 (Fig. 6c) resulted in similar levels of inhibition for both secreted and intracellular particles produced when NS2 was present, as well as for intracellular virions produced in its absence. Thus, intracellular NS2-deficient HCV-LP enter and productively pseudo-infect Huh7 cells via an analogous route to authentic HCV.

### Context-dependent formation of intracellular particles in the absence of NS2

**Fig. 7.**
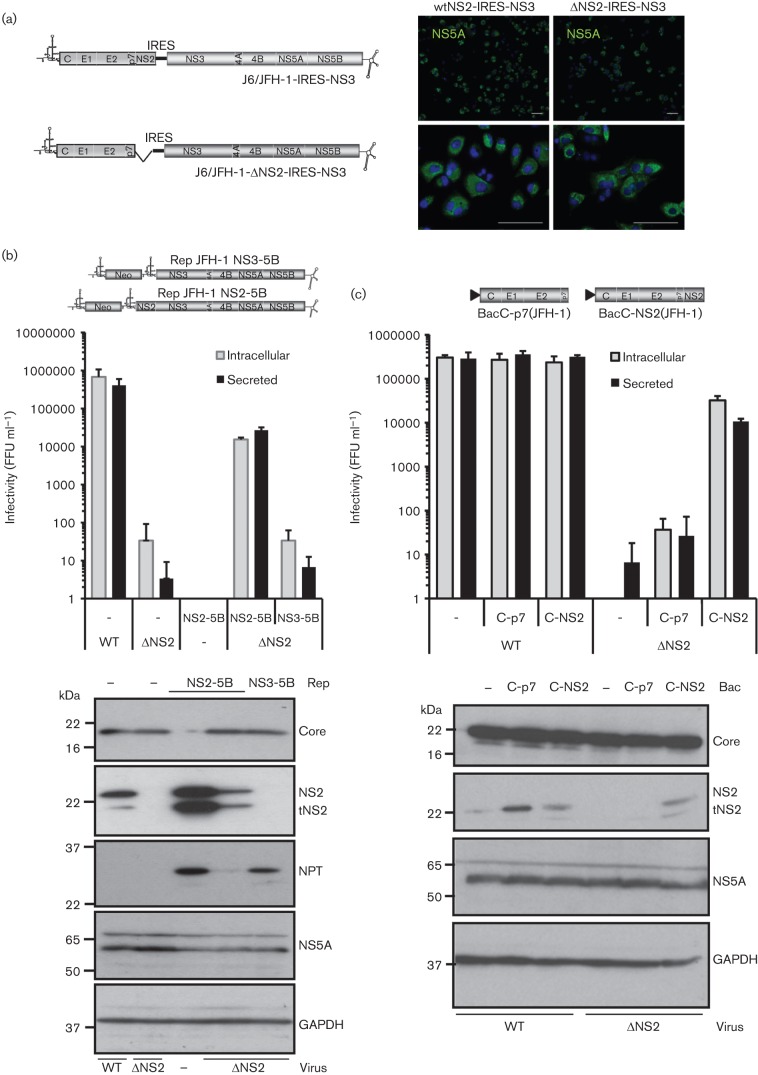
*Trans*-complementation of NS2-deleted HCV using baculovirus-expressed structural proteins and NS2. (a) Schematic of chimeric HCV with NS2/3 expression separated by an IRES, incorporating deletion of the complete NS2 region (Jones *et al.*, 2007). (b) Rescue of NS2-deleted HCV using subgenomic replicon RNAs (full-length : replicon ~1 : 1 molar ratio). Infectivity determined by focus-forming assay at 72 h post-electroporation, protein expression in producer cell lysates assessed by Western blot utilizing antibodies as indicated. (c) As for (b), but with rescue provided by baculoviruses either expressing or lacking NS2 alongside structural proteins.

**Fig. 8.**
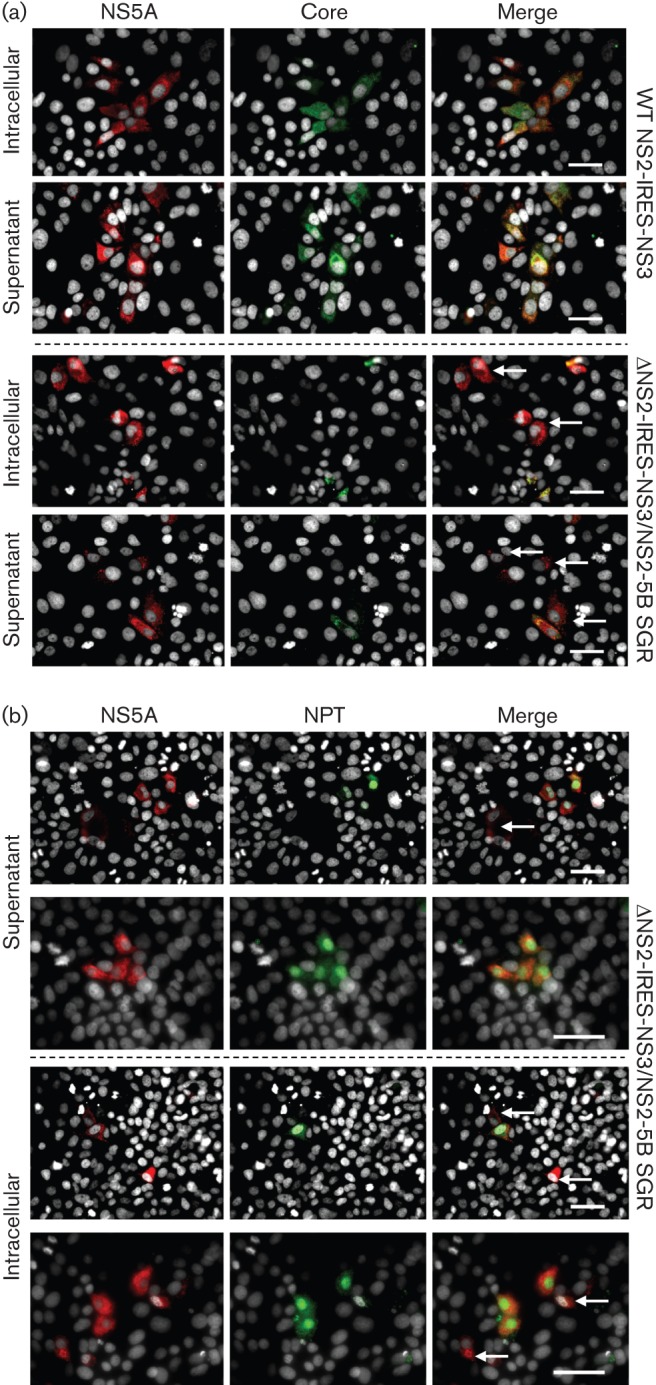
Characterization of HCV RNAs delivered to naive Huh7 cells following replicon-mediated rescue of NS2-deleted HCV. (a) Immunofluorescence staining of infected naive Huh7 cells for marker full-length (core, NS5A) RNA expression. Infectious compartments used to infect cells are as indicated. Top panel shows NS2-positive chimeric virus as a control; lower panel shows infection following rescue with helper replicon RNA. (b) As for (a), but staining of rescue experiments for replicon RNAs (NPT, NS5A).

Our observation that NS2 is not required for the generation of pseudo-infectious intracellular HCV-LP notably differs from published studies where infectivity was absent from bicistronic HCV expressing structural proteins (C-p7) in cistron 1 and the viral replicase (NS3-5B) in cistron 2. It was therefore important to reproduce these data to verify that, in our hands, similar defects in the production of HCV-LP were observed. Consistent with previous reports, transfection of J6/JFH-1-ΔNS2-IRES-NS3 (Fig. 7a) into Huh7 cells failed to generate infectious intracellular or secreted particles, in contrast to its infectious full-length genome counterpart (Fig. 7b, c). Reassuringly, provision of NS2 *in trans*, either via co-transfection with an NS2-5B JFH-1 replicon RNA (Fig. 7b), or BacC-NS2(JFH-1) transduction (Fig. 7c), resulted in the formation of infectious J6/JFH-1-ΔNS2-IRES-NS3 particles. Given the similar efficiency of complementation achieved using these two systems, we conclude that baculovirus transduction does not perturb the cellular machinery in a manner that might interfere with the assembly process. This was confirmed by the observation that BacC-p7(JFH-1) transduction was unable to restore particle production to J6/JFH-1-ΔNS2-IRES-NS3 transfected cells (Fig. 7c). Our results therefore support that intracellular HCV-LP particle production in the absence of NS2 is a genuine phenomenon, but is dictated by the context in which the RNA to be packaged expresses viral proteins. Finally, we assessed which RNAs were encapsidated into newly formed particles during *trans*-complementation assays by staining infected cells for NS5A, core and neomycin phosphotransferase (NPT). As seen for NS5A *trans*-complementation experiments (Appel *et al.*, 2008), infected cells contained both chimeric HCV as well as subgenomic replicon RNAs, as evidenced by a lack of core staining (Fig. 8a) and the presence of NPT (Fig. 8b) within NS5A-positive foci (white arrows depict single-labelling cells in each instance).

## Discussion

This study demonstrates that NS2 is not required for the formation of HCV-LP in a bipartite assembly system where baculovirus-expressed HCV structural proteins package subgenomic replicon RNA. This applies to both genotype 1b HCV, and the cell culture infectious JFH-1 isolate which belongs to genotype 2a. However, efficient secretion of HCV-LP from the cell only occurs when NS2 is present in the genotype 2a JFH-1 context. HCV-LP produced in the presence or absence of NS2 appear to share broadly similar characteristics and are equally ‘pseudo-infectious’, as defined by their ability to transfer subgenomic replicon RNAs to naive Huh7 cells in an E2/CD81 dependent fashion. Interestingly, to our knowledge, this is the first demonstration of pseudo-infectious genotype 1b HCV-LP, which was unexpected due to the presence of cell-culture-adaptive mutations within the FK5.1neo replicon that reduce particle formation in a full-length CON-1 context (Pietschmann *et al.*, 2009). This alludes to potential differences in particle production in *cis* vs *trans* encapsidation settings.

Mutations within NS2 have been identified that modulate several processes linked to the production of infectious HCV particles, including interactions with other HCV proteins, targeting to cytoplasmic foci, and, together with p7, influencing the localization of core protein (Boson *et al.*, 2011; Jirasko *et al.*, 2008, 2010; Ma *et al.*, 2011; Popescu *et al.*, 2011; Stapleford & Lindenbach, 2011; Tedbury *et al.*, 2011). The combination of these functions with the loss of intracellular infectivity has led to the hypothesis that NS2 forms an assembly scaffold within cells, co-ordinating early events of virion morphogenesis. However, two studies also support post-assembly roles for NS2 during infectious particle production, namely the identification of non-infectious H77/JFH-1 chimeric particles carrying an NS2 S168A mutation (Yi *et al.*, 2009), and the generation of N-terminal NS2 point mutants with defects in particle egress (de la Fuente *et al.*, 2013). These studies were carried out primarily in the context of bicistronic full-length HCV clones, where NS2 and NS3 are separated by an IRES and deletion of NS2 abrogates infectivity in all compartments (Jones *et al.*, 2007). However, omission of NS2 from our bipartite system resembles the scenario seen for the N-terminal mutants, where secreted, but not intracellular infectivity is significantly reduced. The mechanism by which *trans*-encapsidation bypasses the requirement for NS2 at an early stage remains unknown, yet seems unlikely to involve overexpression artefacts as protein levels were similar to those observed following electroporation of full-length JFH-1 RNA and baculovirus-expressed proteins were able to rescue NS2-deleted full-length bicistronic HCV *in trans*.

It is interesting to note that bicistronic viruses such as J6/JFH-1-(ΔNS2)-IRES-NS3 differ from subgenomic replicons by expression of the HCV structural proteins, C-p7, in place of luciferase or NPT in the first cistron. Thus, when C-p7 is expressed from a replicating, NS2-deleted HCV RNA, which would otherwise ultimately be destined to be packaged, this appears to prevent the ability of those same structural proteins expressed *in trans* to rescue particle production (see Fig. 7c). This does not appear to be due to the availability or accessibility of C-p7 within these cells as provision of NS2 leads to *trans*-encapsidation of replicon RNAs (Fig. 8). Accordingly, our study shows that separation of C-p7 expression from replicating NS2-deleted RNAs allows particle formation to proceed, and reveals a late-stage NS2 defect during secretion, which has also been observed in mutagenesis studies (de la Fuente *et al.*, 2013; Yi *et al.*, 2009). Interestingly, neither the origin of NS2 (replicon/bicistronic virus/baculovirus), nor limited expression levels [e.g. BacC-NS2(JFH-1)] appear to adversely affect its ability to rescue particle production/secretion in either scenario. This lack of positional dependence may relate to the initial widespread cytoplasmic distribution of NS2 at early times post-infection/transfection, with targeting to foci occurring much later (Jirasko *et al.*, 2010). Taken together, these observations support the notion that distinct pools of replicated/translated/packaged HCV RNA might exist within the cell, and that NS2 may regulate their composition via its multiple protein–protein interactions, in particular the HCV RNA-binding protein, NS5A. Accordingly, NS5A *trans*-complementation studies discriminate between requirements for particle assembly and replication (Herod *et al.*, 2014), and the pharmacokinetics of the NS5Ai, Daclatasvir, support distinct RNA pools for packaging and replication (McGivern *et al.*, 2014).

The *trans*-interaction between NS2 and p7 appears to be integral to the function of both proteins during particle production. This interaction results in p7-mediated targeting of NS2 to punctae (Jirasko *et al.*, 2008, 2010; Ma *et al.*, 2011; Popescu *et al.*, 2011; Tedbury *et al.*, 2011), which may also influence NS2 stability; several mutations that disrupt p7/NS2 interactions also increase the abundance of tNS2 within cells (Jirasko *et al.*, 2008, 2010) and we also observed this to occur where NS2 was expressed separately from C-p7. The precise nature of tNS2 is uncertain, but proteasomal degradation of NS2 is thought to be mediated by casein kinase(II)-mediated phosphorylation of Ser168 (Franck *et al.*, 2005). However, the effects of S168A mutations appear to be genotype-dependent, abrogating infectivity in H77/JFH-1 but not JFH-1 (Yi *et al.*, 2009), although both NS2 localization and its NS5A interaction are disrupted in the latter case (Tedbury *et al.*, 2011). However, localization of NS5A to punctae is unaffected by mutations disrupting p7/NS2 interactions, suggesting that NS2 does not drive punctae formation (Popescu *et al.*, 2011; Tedbury *et al.*, 2011). H77/JFH-1-S168A viruses in the above study also assembled non-infectious particles, consistent with our observation that C-p7 alone is sufficient to assemble virions within Huh7 cells. Combined with NS2 point mutations identified as being specific to particle secretion rather than early events (de la Fuente *et al.*, 2013), the function of this protein appears complex, context-dependent and largely undefined. However, combinations of *cis* and *trans* systems may provide further insight into how this protein regulates both particle formation and release.

Regarding a potential secretory role, Ser 168 phosphorylation is thought to mediate NS2 ubiquitylation, targeting it for proteasomal degradation. However, mutagenesis of NS2 lysine residues affected infectious particle production, rather than NS2 stability (Welbourn *et al.*, 2009). One alternative outcome of protein ubiquitylation is to recruit protein cargo into the endosomal sorting complex required for transport, or ‘ESCRT’ pathway (Babst *et al.*, 2002a, b; Katzmann *et al.*, 2001), which we and others have shown to influence the secretion of infectious HCV particles (Ariumi *et al.*, 2011; Corless *et al.*, 2010). ESCRT factors are critical for the secretion of many enveloped viruses, mediating final membrane scission events during budding. Whilst the involvement of NS2 in such processes is unknown, it is interesting to note that mutations of the p7 basic loop region have recently been shown to disrupt particle envelopment (Gentzsch *et al.*, 2013). However, such p7 mutations also disrupt NS2 stability and localization via effects on E2-p7-NS2 precursors (Bentham *et al.*, 2013; Steinmann *et al.*, 2007; Tedbury *et al.*, 2011). It is therefore possible that envelopment defects were in part caused by disruption of an NS2-ESCRT interaction, explaining its late-acting functions identified both herein and by others (de la Fuente *et al.*, 2013).

In summary, we have exploited a robust *trans*-encapsidation system to demonstrate differential requirements for NS2 during infectious particle production. Detailed comparison between bicistronic full-length and *trans*-encapsidation systems may provide further insight into the seemingly context-dependent, complex role played by this protein during the HCV life cycle.

## Methods

### 

#### Plasmids, viral clones and recombinant baculoviruses.

pJFH-1 (Wakita *et al.*, 2005), pJ6/JFH-1WT/ΔNS2-IRES-NS3 (Jones *et al.*, 2007), pFK5.1neo (Krieger *et al.*, 2001) and subgenomic JFH-1 replicons encoding p7-NS5B and NS2-NS5B (Tedbury *et al.*, 2011) have been described previously. Baculoviruses expressing JFH-1 C-p7 and C-NS2 have also been described previously (Adair *et al.*, 2009). Passage 1 (P1) stocks were prepared by identical methods, but P3 stocks were generated commercially by Oxford Expression Technologies. Baculoviruses expressing genotype 1b C-p7/E1-p7 were generated identically to those expressing JFH-1 structural proteins using pCV-J4L6S (Yanagi *et al.*, 1998) as a template and specific primers. Details are available upon request.

#### Cell and virus culture.

Huh7 cells were maintained and transfected with replicon and wt full-length JFH-1-derived RNAs as described previously (Griffin *et al.*, 2008). Huh7 cells harbouring the GT1b replicon (FK5.1neo) were maintained in Dulbecco’s minimal essential medium supplemented with 250 *μ*g G418 ml^−1^ (Life Technologies). For baculovirus transduction, Huh7 FK5.1neo cells were inoculated with appropriate GT1b P3 baculovirus (or control wt-LacZ) at an m.o.i. of 100–200 p.f.u. per cell and incubated for 96 h. Cell lysates were prepared by sonication (or freeze–thaw for genotype 2a) in TNE buffer (50 mM Tris, 50 mM NaCl, 0.5 mM EDTA, pH 7.5), supplemented with protease inhibitors, then clarified by low speed centrifugation (15 min, 900 ***g***). For colony formation assays, naive Huh7 cells were inoculated directly with clarified lysates as described previously (Foster *et al.*, 2011), and pseudo-infected cells were selected using 1 mg G418 ml^−1^, final concentration. G418-resistant colonies became visible after 2 weeks, at which point they were fixed in methanol and stained with Coomassie blue. Baculovirus (m.o.i. 10) transduction of JFH-1 replicons was at 18 h post electroporation and cells/supernatants were harvested at 72 h post-transfection. Supernatant HCV-LPs (or wt virus) were clarified at 1000 ***g*** and quantified by focus-forming assay. Intracellular virus was prepared by four cycles of freeze/thaw lysis in PBS (250 µl), as described previously (Foster *et al.*, 2011).

#### Protein analysis.

Antibodies used in this study were as follows: rabbit anti-core polyclonal serum (308 or R412), mouse anti-E2 monoclonal [AP33 and ALP98 (Flint *et al.*, 1999; Owsianka *et al.*, 2001)], rabbit polyclonal NS2 antiserum (Jirasko *et al.*, 2010), sheep anti-NS5A polyclonal serum (Macdonald *et al.*, 2003), mouse anti-GAPDH monoclonal (6C5, Abcam), mouse anti-neomycin phosphotransferase 2 (4B4D1/ab60018, Abcam), mouse anti-CD81 (human) (BD Pharmingen), and rabbit-anti-MBP (NEB). Appropriate HRP-conjugated secondary antibodies were obtained from Sigma. Alexa-fluor conjugated secondary antibodies were obtained from Invitrogen. For Western analysis, Huh7 cells were lysed in enriched broth culture (EBC) lysis buffer, then subjected to SDS-PAGE and Western analysis as described previously (Griffin *et al.*, 2008). AP33 was used in the antibody neutralization assay along with an appropriate isotype control.

Recombinant CD81 large extracellular loop (LEL) fused to maltose binding protein (MBP–CD81) was expressed and purified as described by Chan-Fook *et al.* (2000). MBP–CD81 fusion protein (3.75 µg) was incubated with 450 µl cell lysate samples at 4 °C for 1 h. Amylose resin (100 µl; NEB) as a 1 : 5 slurry in TNE buffer [100 mM Tris/HCl (pH 7.4), 150 mM NaCl, 5 mM EDTA] was added, and further incubated for 4 h. The resin was extensively washed in TNE buffer, boiled in sample loading buffer, clarified, and subjected to SDS-PAGE prior to Western blotting. For co-immunoprecipitation of E2 and core from the GT1b *trans*-encapsidation experiments, cleared cell lysate was incubated with specified antibody and complexes were captured on protein-G agarose beads (Life Technologies). After incubation (4 °C, 4 h) beads were repeatedly washed in TNE buffer, before the proteins were released by boiling in SDS sample buffer. Proteins were analysed by SDS-PAGE and Western blot. NP-40 (0.1 % final concentration) was added to the cleared lysate and included in subsequent steps as indicated.

#### Detection of HCV RNA.

Detection of HCV-specific RNA (5′ UTR, primer sequences available on request) from HCV-LP concentrated by immunoprecipitation/pull-down or from within gradient fractions was achieved by extraction with Trizol reagent (Gibco Life Technologies), followed by treatment with DNase I (RQI, Promega) prior to RT-PCR using Superscript reverse transcriptase (Gibco Life Technogies) following the manufacturer’s instructions.

#### Purification of secreted and intracellular HCV-LP.

For intracellular HCV-LP, clarified cell lysates were first diluted to 10 ml with PBS and then partially purified by centrifugation at 150 000 ***g*** through a 2 ml 20 % sucrose cushion (w/v in PBS) for 4 h at 4 °C in a Sorvall TH-641 rotor. Extracellular HCV-LP were purified identically from 10 ml clarified culture supernatant. The resulting pellet was resuspended in 250 µl PBS and samples were taken for titre determination and Western blot analysis (input on Fig. 6a). Genotype 1b HCV-LP cushion-purified lysates were layered over a 20–60 % (w/w) sucrose gradient in PBS (pH 7.4) and centrifuged in a Sorvall OTD65B (TH-641 rotor) for 22 h at 150 000 ***g***. Twenty fractions were collected from the top and analysed by SDS-PAGE; a small portion of each was retained for TEM analysis. JFH-1 replicon *trans*-encapsidated particles and/or wt JFH-1 virus were purified on a preformed 12 ml iodixinol (10–40 %, w/v, in PBS) gradient and centrifuged in a Sorvall TH-641 rotor for 17 h at 80 000 ***g***. Twenty-two equal fractions were taken; 10 µl of each was titrated on naive Huh7 cells by focus-forming assays. Alternatively, samples of each fraction were mixed with an equal volume of 4 % paraformaldehyde/PBS prior to TEM analysis. Ice-cold methanol was added to the remainder of each fraction (3 : 1) and proteins precipitated at −20 °C for 18 h. Precipitated proteins were recovered by centrifugation (13 000 r.p.m., bench top microfuge, 4 °C), supernatants removed and pellets resuspended in SDS-PAGE loading buffer, prior to Western blot analysis.

#### Electron microscopy.

Selected gradient fractions were prepared for TEM by a uranyl acetate staining procedure on nickel carbon-coated grids. Selected fractions (3–10 µl) were applied to Formvar-carbon 300 mesh nickel grids (Agar Scientific), semi-dried and repeatedly washed with PBS prior to staining with 2 % uranyl acetate solution for 20 s. The grids were washed and examined using a JEOL 1200ex transmission electron microscope operating at 80 kV. Thin-section electron microscopy was carried out according to the method of Shimizu *et al.* (1996). Briefly, Huh7 cells containing the FK5.1neo replicon were transduced with BacC-p7(1b) or BacE1-p7(1b) for 72 h at 37 °C. Cultured cells were fixed in 2.5 % glutaraldehyde, post-fixed with 1 % osmium tetroxide, treated with 2 % uranyl acetate, dehydrated in ethanol, and embedded in araldite-propylene oxide. Ultrathin sections were stained with lead citrate.

## References

[r1] Adair R. Patel A. H. Corless L. Griffin S. Rowlands D. J. McCormick C. J. **(**2009**).** Expression of hepatitis C virus (HCV) structural proteins *in trans* facilitates encapsidation and transmission of HCV subgenomic RNA. J Gen Virol 90, 833–842. 10.1099/vir.2008.006049-0 19223490

[r2] Appel N. Zayas M. Miller S. Krijnse-Locker J. Schaller T. Friebe P. Kallis S. Engel U. Bartenschlager R. **(**2008**).** Essential role of domain III of nonstructural protein 5A for hepatitis C virus infectious particle assembly. PLoS Pathog 4, e1000035. 10.1371/journal.ppat.1000035 18369481 PMC2268006

[r3] Ariumi Y. Kuroki M. Maki M. Ikeda M. Dansako H. Wakita T. Kato N. **(**2011**).** The ESCRT system is required for hepatitis C virus production. PLoS ONE 6, e14517. 10.1371/journal.pone.0014517 21264300 PMC3019154

[r4] Babst M. Katzmann D. J. Estepa-Sabal E. J. Meerloo T. Emr S. D. **(**2002a**).** Escrt-III: an endosome-associated heterooligomeric protein complex required for mvb sorting. Dev Cell 3, 271–282. 10.1016/S1534-5807(02)00220-4 12194857

[r5] Babst M. Katzmann D. J. Snyder W. B. Wendland B. Emr S. D. **(**2002b**).** Endosome-associated complex, ESCRT-II, recruits transport machinery for protein sorting at the multivesicular body. Dev Cell 3, 283–289. 10.1016/S1534-5807(02)00219-8 12194858

[r6] Baumert T. F. Ito S. Wong D. T. Liang T. J. **(**1998**).** Hepatitis C virus structural proteins assemble into viruslike particles in insect cells. J Virol 72, 3827–3836. 9557666 10.1128/jvi.72.5.3827-3836.1998 PMC109606

[r7] Bentham M. J. Foster T. L. McCormick C. Griffin S. **(**2013**).** Mutations in hepatitis C virus p7 reduce both the egress and infectivity of assembled particles via impaired proton channel function. J Gen Virol 94, 2236–2248. 10.1099/vir.0.054338-0 23907396

[r8] Boson B. Granio O. Bartenschlager R. Cosset F. L. **(**2011**).** A concerted action of hepatitis C virus p7 and nonstructural protein 2 regulates core localization at the endoplasmic reticulum and virus assembly. PLoS Pathog 7, e1002144. 10.1371/journal.ppat.1002144 21814513 PMC3141040

[r10] Chan-Fook C. Jiang W. R. Clarke B. E. Zitzmann N. Maidens C. McKeating J. A. Jones I. M. **(**2000**).** Hepatitis C virus glycoprotein E2 binding to CD81: the role of E1E2 cleavage and protein glycosylation in bioactivity. Virology 273, 60–66. 10.1006/viro.2000.0407 10891408

[r11] Corless L. Crump C. M. Griffin S. D. Harris M. **(**2010**).** Vps4 and the ESCRT-III complex are required for the release of infectious hepatitis C virus particles. J Gen Virol 91, 362–372. 10.1099/vir.0.017285-0 19828764 PMC7615705

[r12] de la Fuente C. Goodman Z. Rice C. M. **(**2013**).** Genetic and functional characterization of the N-terminal region of the hepatitis C virus NS2 protein. J Virol 87, 4130–4145. 10.1128/JVI.03174-12 23408609 PMC3624385

[r13] Flint M. Thomas J. M. Maidens C. M. Shotton C. Levy S. Barclay W. S. McKeating J. A. **(**1999**).** Functional analysis of cell surface-expressed hepatitis C virus E2 glycoprotein. J Virol 73, 6782–6790. 10400776 10.1128/jvi.73.8.6782-6790.1999 PMC112763

[r14] Foster T. L. Verow M. Wozniak A. L. Bentham M. J. Thompson J. Atkins E. Weinman S. A. Fishwick C. Foster R. **& other authors (**2011**).** Resistance mutations define specific antiviral effects for inhibitors of the hepatitis C virus p7 ion channel. Hepatology 54, 79–90. 10.1002/hep.24371 21520195

[r15] Franck N. Le Seyec J. Guguen-Guillouzo C. Erdtmann L. **(**2005**).** Hepatitis C virus NS2 protein is phosphorylated by the protein kinase CK2 and targeted for degradation to the proteasome. J Virol 79, 2700–2708. 10.1128/JVI.79.5.2700-2708.2005 15708989 PMC548468

[r16] Gentzsch J. Brohm C. Steinmann E. Friesland M. Menzel N. Vieyres G. Perin P. M. Frentzen A. Kaderali L. Pietschmann T. **(**2013**).** Hepatitis C virus p7 is critical for capsid assembly and envelopment. PLoS Pathog 9, e1003355. 10.1371/journal.ppat.1003355 23658526 PMC3642076

[r17] Griffin S. Stgelais C. Owsianka A. M. Patel A. H. Rowlands D. Harris M. **(**2008**).** Genotype-dependent sensitivity of hepatitis C virus to inhibitors of the p7 ion channel. Hepatology 48, 1779–1790. 10.1002/hep.22555 18828153 PMC7615706

[r18] Herod M. R. Schregel V. Hinds C. Liu M. McLauchlan J. McCormick C. J. Sandri-Goldin R. M. **(**2014**).** Genetic complementation of hepatitis C virus nonstructural protein functions associated with replication exhibits requirements that differ from those for virion assembly. J Virol 88, 2748–2762. 10.1128/JVI.03588-13 24352463 PMC3958097

[r19] Ishii K. Murakami K. Hmwe S. S. Zhang B. Li J. Shirakura M. Morikawa K. Suzuki R. Miyamura T. **& other authors (**2008**).** Trans-encapsidation of hepatitis C virus subgenomic replicon RNA with viral structure proteins. Biochem Biophys Res Commun 371, 446–450. 10.1016/j.bbrc.2008.04.110 18445476

[r20] Jirasko V. Montserret R. Appel N. Janvier A. Eustachi L. Brohm C. Steinmann E. Pietschmann T. Penin F. Bartenschlager R. **(**2008**).** Structural and functional characterization of nonstructural protein 2 for its role in hepatitis C virus assembly. J Biol Chem 283, 28546–28562. 10.1074/jbc.M803981200 18644781 PMC2661407

[r21] Jirasko V. Montserret R. Lee J. Y. Gouttenoire J. Moradpour D. Penin F. Bartenschlager R. **(**2010**).** Structural and functional studies of nonstructural protein 2 of the hepatitis C virus reveal its key role as organizer of virion assembly. PLoS Pathog 6, e1001233. 10.1371/journal.ppat.1001233 21187906 PMC3002993

[r22] Jones C. T. Murray C. L. Eastman D. K. Tassello J. Rice C. M. **(**2007**).** Hepatitis C virus p7 and NS2 proteins are essential for production of infectious virus. J Virol 81, 8374–8383. 10.1128/JVI.00690-07 17537845 PMC1951341

[r23] Kato T. Date T. Miyamoto M. Furusaka A. Tokushige K. Mizokami M. Wakita T. **(**2003**).** Efficient replication of the genotype 2a hepatitis C virus subgenomic replicon. Gastroenterology 125, 1808–1817. 10.1053/j.gastro.2003.09.023 14724833

[r24] Katzmann D. J. Babst M. Emr S. D. **(**2001**).** Ubiquitin-dependent sorting into the multivesicular body pathway requires the function of a conserved endosomal protein sorting complex, ESCRT-I. Cell 106, 145–155. 10.1016/S0092-8674(01)00434-2 11511343

[r25] Krieger N. Lohmann V. Bartenschlager R. **(**2001**).** Enhancement of hepatitis C virus RNA replication by cell culture-adaptive mutations. J Virol 75, 4614–4624. 10.1128/JVI.75.10.4614-4624.2001 11312331 PMC114214

[r26] Kunkel M. Lorinczi M. Rijnbrand R. Lemon S. M. Watowich S. J. **(**2001**).** Self-assembly of nucleocapsid-like particles from recombinant hepatitis C virus core protein. J Virol 75, 2119–2129. 10.1128/JVI.75.5.2119-2129.2001 11160716 PMC114796

[r27] Ma Y. Anantpadma M. Timpe J. M. Shanmugam S. Singh S. M. Lemon S. M. Yi M. **(**2011**).** Hepatitis C virus NS2 protein serves as a scaffold for virus assembly by interacting with both structural and nonstructural proteins. J Virol 85, 86–97. 10.1128/JVI.01070-10 20962101 PMC3014171

[r28] Macdonald A. Crowder K. Street A. McCormick C. Saksela K. Harris M. **(**2003**).** The hepatitis C virus non-structural NS5A protein inhibits activating protein-1 function by perturbing ras-ERK pathway signaling. J Biol Chem 278, 17775–17784. 10.1074/jbc.M210900200 12621033

[r29] McGivern D. R. Masaki T. Williford S. Ingravallo P. Feng Z. Lahser F. Asante-Appiah E. Neddermann P. De Francesco R. **& other authors (**2014**).** Kinetic analyses reveal potent and early blockade of hepatitis C virus assembly by NS5A inhibitors. Gastroenterology 147, 453, e7. 10.1074/jbc.M210900200 24768676 PMC4107048

[r30] Owsianka A. Clayton R. F. Loomis-Price L. D. McKeating J. A. Patel A. H. **(**2001**).** Functional analysis of hepatitis C virus E2 glycoproteins and virus-like particles reveals structural dissimilarities between different forms of E2. J Gen Virol 82, 1877–1883. 11457993 10.1099/0022-1317-82-8-1877

[r31] Pietschmann T. Zayas M. Meuleman P. Long G. Appel N. Koutsoudakis G. Kallis S. Leroux-Roels G. Lohmann V. Bartenschlager R. **(**2009**).** Production of infectious genotype 1b virus particles in cell culture and impairment by replication enhancing mutations. PLoS Pathog 5, e1000475. 10.1371/journal.ppat.1000475 19521536 PMC2691593

[r32] Popescu C. I. Callens N. Trinel D. Roingeard P. Moradpour D. Descamps V. Duverlie G. Penin F. Héliot L. **& other authors (**2011**).** NS2 protein of hepatitis C virus interacts with structural and non-structural proteins towards virus assembly. PLoS Pathog 7, e1001278. 10.1371/journal.ppat.1001278 21347350 PMC3037360

[r33] Saunier B. Triyatni M. Ulianich L. Maruvada P. Yen P. Kohn L. D. **(**2003**).** Role of the asialoglycoprotein receptor in binding and entry of hepatitis C virus structural proteins in cultured human hepatocytes. J Virol 77, 546–559. 10.1128/JVI.77.1.546-559.2003 12477859 PMC140572

[r34] Shimizu Y. K. Feinstone S. M. Kohara M. Purcell R. H. Yoshikura H. **(**1996**).** Hepatitis C virus: detection of intracellular virus particles by electron microscopy. Hepatology 23, 205–209. 10.1002/hep.510230202 8591842

[r35] Stapleford K. A. Lindenbach B. D. **(**2011**).** Hepatitis C virus NS2 coordinates virus particle assembly through physical interactions with the E1-E2 glycoprotein and NS3-NS4A enzyme complexes. J Virol 85, 1706–1717. 10.1128/JVI.02268-10 21147927 PMC3028914

[r36] Steinmann E. Penin F. Kallis S. Patel A. H. Bartenschlager R. Pietschmann T. **(**2007**).** Hepatitis C virus p7 protein is crucial for assembly and release of infectious virions. PLoS Pathog 3, e103. 10.1371/journal.ppat.0030103 17658949 PMC1924870

[r37] Steinmann E. Brohm C. Kallis S. Bartenschlager R. Pietschmann T. **(**2008**).** Efficient trans-encapsidation of hepatitis C virus RNAs into infectious virus-like particles. J Virol 82, 7034–7046. 10.1128/JVI.00118-08 18480457 PMC2446957

[r38] Tedbury P. Welbourn S. Pause A. King B. Griffin S. Harris M. **(**2011**).** The subcellular localization of the hepatitis C virus non-structural protein NS2 is regulated by an ion channel-independent function of the p7 protein. J Gen Virol 92, 819–830. 10.1099/vir.0.027441-0 21177929 PMC3133701

[r39] Triyatni M. Saunier B. Maruvada P. Davis A. R. Ulianich L. Heller T. Patel A. Kohn L. D. Liang T. J. **(**2002**).** Interaction of hepatitis C virus-like particles and cells: a model system for studying viral binding and entry. J Virol 76, 9335–9344. 10.1128/JVI.76.18.9335-9344.2002 12186916 PMC136469

[r40] Wakita T. Pietschmann T. Kato T. Date T. Miyamoto M. Zhao Z. Murthy K. Habermann A. Kräusslich H. G. **& other authors (**2005**).** Production of infectious hepatitis C virus in tissue culture from a cloned viral genome. Nat Med 11, 791–796. 10.1038/nm1268 15951748 PMC2918402

[r41] Welbourn S. Jirasko V. Breton V. Reiss S. Penin F. Bartenschlager R. Pause A. **(**2009**).** Investigation of a role for lysine residues in non-structural proteins 2 and 2/3 of the hepatitis C virus for their degradation and virus assembly. J Gen Virol 90, 1071–1080. 10.1099/vir.0.009944-0 19264595

[r42] Wellnitz S. Klumpp B. Barth H. Ito S. Depla E. Dubuisson J. Blum H. E. Baumert T. F. **(**2002**).** Binding of hepatitis C virus-like particles derived from infectious clone H77C to defined human cell lines. J Virol 76, 1181–1193. 10.1128/JVI.76.3.1181-1193.2002 11773394 PMC135804

[r43] Xiang J. Wünschmann S. George S. L. Klinzman D. Schmidt W. N. LaBrecque D. R. Stapleton J. T. **(**2002**).** Recombinant hepatitis C virus-like particles expressed by baculovirus: utility in cell-binding and antibody detection assays. J Med Virol 68, 537–543. 10.1002/jmv.10237 12376962

[r44] Yanagi M. St Claire M. Shapiro M. Emerson S. U. Purcell R. H. Bukh J. **(**1998**).** Transcripts of a chimeric cDNA clone of hepatitis C virus genotype 1b are infectious *in vivo*. Virology 244, 161–172. 10.1006/viro.1998.9092 9581788

[r45] Yi M. Ma Y. Yates J. Lemon S. M. **(**2009**).** Trans-complementation of an NS2 defect in a late step in hepatitis C virus (HCV) particle assembly and maturation. PLoS Pathog 5, e1000403. 10.1371/journal.ppat.1000403 19412343 PMC2669722

